# Interactions between xenoestrogens and ketoconazole on hepatic CYP1A and CYP3A, in juvenile Atlantic cod (*Gadus morhua*)

**DOI:** 10.1186/1476-5926-4-2

**Published:** 2005-02-08

**Authors:** Linda Hasselberg, Bjørn E Grøsvik, Anders Goksøyr, Malin C Celander

**Affiliations:** 1 Department of Zoophysiology, Göteborg University, Box 463, SE 405 30 Göteborg, Sweden; 2 Department of Molecular Biology, HIB, University of Bergen, N 5020 Bergen, Norway; 3 Biosense Laboratories AS, N-5008, Bergen, Norway

## Abstract

**Background:**

Xenoestrogens and antifungal azoles probably share a common route of metabolism, through hepatic cytochrome P450 (CYP) enzymes. Chemical interactions with metabolic pathways may affect clearance of both xenobiotics and endobiotics. This study was carried out to identify possible chemical interactions by those substances on CYP1A and CYP3A, in Atlantic cod liver. We investigated effects of two xenoestrogens (nonylphenol and ethynylestradiol) and of the model imidazole ketoconazole, alone and in combination.

**Results:**

Treatment with ketoconazole resulted in 60% increase in CYP1A-mediated ethoxyresorufin-*O*-deethylase (EROD) activity. Treatment with nonylphenol resulted in 40% reduction of CYP1A activity. Combined exposure to ketoconazole and nonylphenol resulted in 70% induction of CYP1A activities and 93% increase in CYP1A protein levels. Ketoconazole and nonylphenol alone or in combination had no effect on CYP3A expression, as analyzed by western blots. However, 2-dimensional (2D) gel electrophoresis revealed the presence of two CYP3A-immunoreactive proteins, with a more basic isoform induced by ketoconazole. Treatment with ketoconazole and nonylphenol alone resulted in 54% and 35% reduction of the CYP3A-mediated benzyloxy-4-[trifluoromethyl]-coumarin-*O*-debenzyloxylase (BFCOD) activity. Combined exposure of ketoconazole and nonylphenol resulted in 98% decrease in CYP3A activity. This decrease was greater than the additive effect of each compound alone. *In vitro *studies revealed that ketoconazole was a potent non-competitive inhibitor of both CYP1A and CYP3A activities and that nonylphenol selectively non-competitively inhibited CYP1A activity. Treatment with ethynylestradiol resulted in 46% decrease in CYP3A activity and 22% decrease in protein expression *in vivo*. *In vitro *inhibition studies in liver microsomes showed that ethynylestradiol acted as a non-competitive inhibitor of CYP1A activity and as an uncompetitive inhibitor of CYP3A activity.

**Conclusions:**

Ketoconazole, nonylphenol and ethynylestradiol all interacted with CYP1A and CYP3A activities and protein expression in Atlantic cod. However, mechanisms of interactions on CYP1A and CYP3A differ between theses substances and combined exposure had different effects than exposure to single compounds. Thus, CYP1A and CYP3A mediated clearance may be impaired in situations of mixed exposure to those types of compounds.

## Background

A great challenge in pharmacology and toxicology is to understand the molecular mechanisms behind how mixtures of compounds affect living organisms. This study focuses on two classes of substances, imidazoles and xenoestrogens, and how these chemicals alone and in combination affect hepatic drug-metabolizing hepatic cytochrome P450 (CYP) enzymes – specifically, CYP1A and CYP3A enzymes, in juvenile Atlantic cod (*Gadus morhua*).

Imidazoles and triazoles are used as fungicides both clinically as well as in horticulture and agriculture, posing a potential threat to wildlife. The triazole propiconazole has been detected in the aquatic environment [1]. The azole antifungal effect resides in inhibition of CYP51 mediated ergosterol biosynthesis [2]. In addition to disrupting key enzymes in fungus, azoles such as the imidazoles clotrimazole, ketoconazole, miconazole and prochloraz also cause endocrine disruption in vertebrates by inhibition of key enzymes in steroid homeostasis [3-7]. Moreover, these fungicides inhibit drug-metabolizing CYP forms, including members of the CYP1, CYP2 and CYP3 gene families in vertebrates [5,8-13]. Effects on CYP forms may have adverse effects on metabolic clearance of endobiotics and xenobiotics. For example, in a study in fish, pre-exposure to clotrimazole resulted in increased bioaccumulation of the pro-carcinogen benzo [*a*]pyrene in gizzard shad (*Dorosoma cepedianum*) [14].

Xenoestrogens comprise a wide variety of structurally diverse chemicals such as *o,p*-DDT, ethynylestradiol, alkylphenols and bisphenol A. These substances are well-known or supposed to be endocrine disrupting substances in vertebrates and share in common that they activate the estrogen receptor (ER) and thereby elicit estrogenic responses [15-17]. In addition to being estrogenic, these xenoestrogens interact with drug-metabolizing CYP forms, including members of the CYP1A and CYP3A subfamilies in vertebrates [18-22].

Xenoestrogens are continuously released into the environment as a result of various anthropogenic activities. Induction of vitellogenesis in fish is a biomarker routinely used to assess the presence of estrogenic substances in the aquatic environment [23,24]. Induction of CYP1A-mediated ethoxyresorufin-*O*-deethylase (EROD) activity is another established biomarker used to assess exposure to aromatic hydrocarbons. This response proceeds through activation of the aryl hydrocarbon receptor (AHR) by aromatic hydrocarbons including polyaromatic hydrocarbons, and planar polychlorinated biphenyls and dioxins [25]. Some AHR agonists have been shown to be anti-estrogenic and cross-talk between AHR and ER has been suggested in vertebrates [26-33].

In addition to activation of the ER, xenoestrogens also affect other steroid receptors. Nonylphenol up-regulated CYP3A1 gene expression in rat, through activation of the pregnane X receptor (PXR) [34,35]. We previously reported induction of CYP3A and CYP1A protein levels in Atlantic cod exposed to alkylphenols [22].

Azole fungicides induce expression of multiple vertebrate CYP genes including members of the CYP1A, CYP2B and CYP3A subfamilies [8,9,13,36-38]. Clotrimazole activates the ligand-binding domain of the PXR, involved in CYP3A signalling, *in vitro *from several mammalian species and zebra fish (*Danio rerio*) [39]. Both imidazoles and xenoestrogens inhibit drug-metabolizing enzymes, including members of the CYP1A and CYP3A subfamilies in vertebrates [8-13,18,20,22]. Thus, xenoestrogens and imidazoles conceivably share common routes for biotransformation. However, there is a lack of data regarding effects of combined exposure of imidazoles and xenoestrogens on these CYP forms in wildlife. Living organisms usually are exposed to mixtures of different classes of xenobiotics. Conceivably, exposure to mixtures may be more of a health threat than exposure to single compounds, as a result of interactions. Anthropogenic compounds may enter the environment through industrial activities and through the use of pharmaceuticals [40]. Atlantic cod is an economically important species for fishery and a growing aquaculture industry, in addition to its ecological relevancy. Its distribution in the Northern Atlantic and the North Sea makes it vulnerable to effluents from on-shore and off-shore industries and from run-off entering the waters near highly industrialized and urbanized areas.

The rationale of the present study was to identify possible sites of interactions between imidazoles and xenoestrogens. We hypothesise that combined exposure to these compounds may compromise the metabolic clearance not only of these xenobiotics themselves, but also of endobiotics such as circulating steroid hormones that share common routes of metabolism through hepatic CYP1A and CYP3A. Such endocrine disrupting effects may adversely affect the stability of wildlife populations.

The specific aim of our study was to examine interactions between two classes of compounds in livers of Atlantic cod. Thus, we investigated the effects of the model imidazole ketoconazole and of two types of xenoestrogens (nonylphenol and ethynylestradiol), as well as of a mixed exposure to ketoconazole and nonylphenol, on hepatic CYP1A and CYP3A protein expression and catalytic activities, and also on vitellogenesis and plasma levels of sex steroid hormones.

## Results

### *In vivo *effects on CYP1A

**Figure 1:**
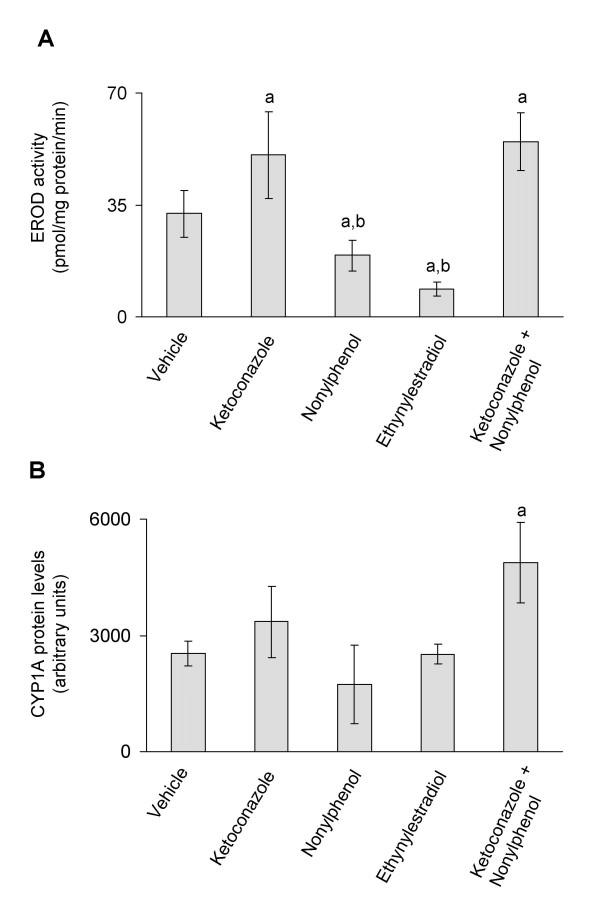
**A) *In vivo *CYP1A enzyme activities (A) and *in vivo *CYP1A protein expression (B)**. CYP1A enzyme activities and protein expression in juvenile Atlantic cod exposed *in vivo *to vehicle (5 ml peanut oil/kg fish), ketoconazole (12 mg/kg fish), nonylphenol (25 mg/kg fish), ethynylestradiol (5 mg/kg fish) and ketoconazole + nonylphenol (12 + 25 mg/kg fish). A) EROD activities. B) CYP1A protein levels analyzed using PAb against rainbow trout CYP1A. Each bar represents mean values of eight to nine fish ± SD; ^a^Significantly different from vehicle treated fish; ^b^Significantly different from ketoconazole+nonylphenol treated fish; *P *< 0.05.

Exposure to ketoconazole (12 mg/kg b.w.) and/or a combination of ketoconazole and nonylphenol (12 mg/kg b.w. + 25 mg/kg b.w.) resulted, respectively, in 159 and 172% average induced increases in CYP1A-mediated EROD activities (Fig. 1A), and in 133 and 193% increases in CYP1A protein levels in Atlantic cod (Fig. 1B). Treatment with nonylphenol (25 mg/kg b.w.) resulted in 41% reduction and ethynylestradiol (5 mg/kg b.w.) resulted in 72% reduction, respectively, of CYP1A activities compared to vehicle treated fish (Fig. 1A). However, when compared to fish exposed to the combination of ketoconazole and nonylphenol, exposure to nonylphenol alone and ethynylestradiol resulted in 65% and 84% decrease in CYP1A activity (Fig. 1A). Exposure to nonylphenol and ethynylestradiol had no effect on CYP1A protein expression (Fig. 1B). The CYP1A protein levels were elevated by 93% in fish exposed to a mixture of ketoconazole and nonylphenol (Fig. 1B).

### *In vivo *effects on CYP3A

**Figure 2:**
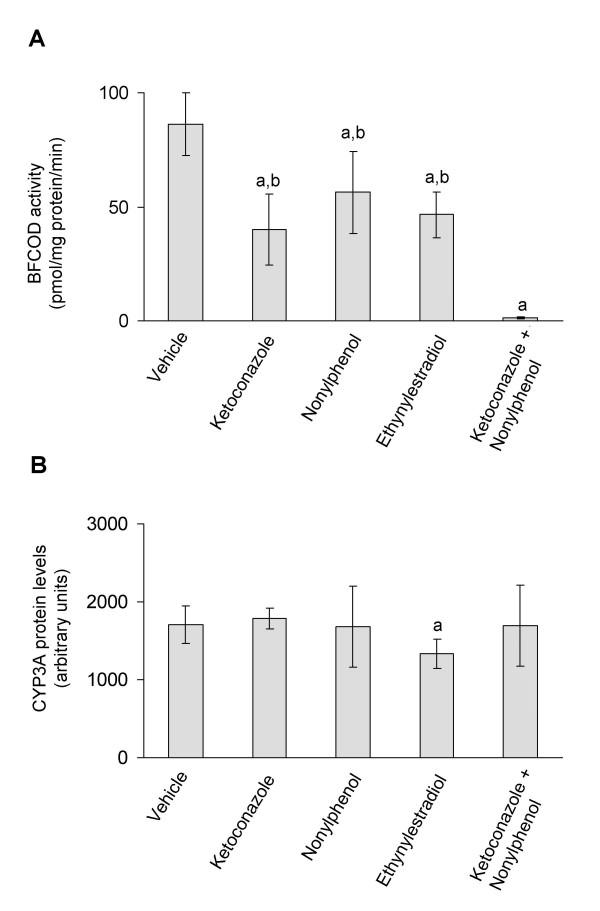
***In vivo *CYP3A enzyme activities (A) and *in vivo *CYP3A protein expression (B)**. CYP3A enzyme activities and protein expression in juvenile Atlantic cod exposed *in vivo *to vehicle (5 ml peanut oil/kg fish), ketoconazole (12 mg/kg fish), nonylphenol (25 mg/kg fish), ethynylestradiol (5 mg/kg fish) and ketoconazole + nonylphenol (12 + 25 mg/kg fish). A) BFCOD activities. B) CYP3A protein levels analyzed using PAb against rainbow trout CYP3A. Each bar represents mean values of eight to nine fish ± SD; ^a^Significantly different from vehicle treated fish; ^b^Significantly different from ketoconazole+nonylphenol treated fish; *P *< 0.05.

Fish exposure to ketoconazole, ethynylestradiol and nonylphenol resulted in decreased CYP3A-mediated benzyloxy-4-[trifluoromethyl]-coumarin-*O*-debenzyloxylase (BFCOD) activities, when compared to vehicle treated fish (Fig. 2A). Furthermore, mixed exposure to ketoconazole and nonylphenol resulted in a 98% decrease in CYP3A activity, which was greater than the additive effects of these two compounds administrated alone (Fig. 2A). Fish exposed to the ketoconazole and nonylphenol mixture displayed significantly reduced CYP3A activities when compared all other treatment groups (Fig. 2A). No effect on CYP3A protein expression was observed in fish treated with ketoconazole and nonylphenol, either alone or in combination (Fig. 2B). However, ethynylestradiol treatment resulted in 22% decrease in CYP3A protein levels (Fig. 2B).

**Figure 3:**
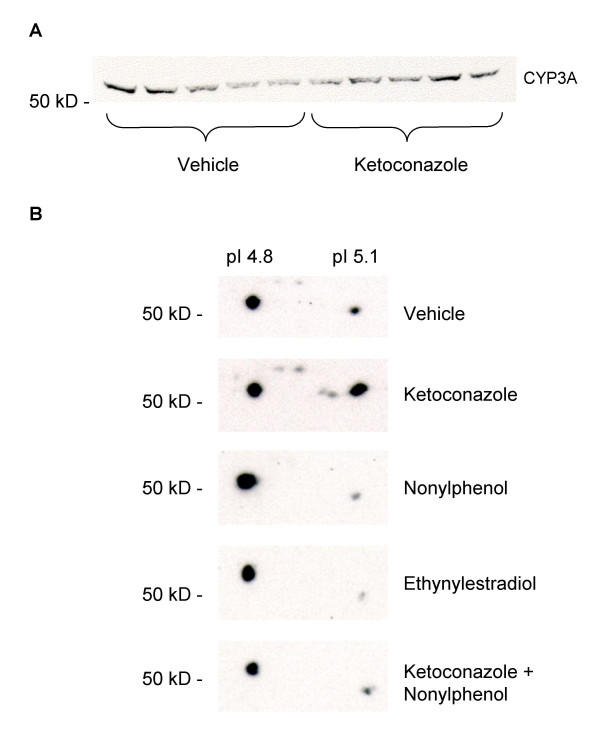
**CYP3A Western blot (A) and CYP3A 2D-immunoblots (B)**. A) Western blot of hepatic microsomal CYP3A proteins in juvenile Atlantic cod treated with vehicle (5 ml peanut oil/kg fish) and ketoconazole (12 mg/kg fish) detected using PAb against rainbow trout CYP3A. B) 2D-gel electrophoresis followed by immunoblotting using PAb against rainbow trout CYP3A. Each blot represent pooled liver microsomes of eight to nine fish for each treatment; vehicle (5 ml peanut oil/kg fish), ketoconazole (12 mg/kg fish), nonylphenol (25 mg/kg fish), ethynylestradiol (5 mg/kg fish), ketoconazole + nonylphenol (12 + 25 mg/kg fish).

Western blot analyses of CYP3A proteins using PAb against rainbow trout CYP3A revealed the presence of one CYP3A immunoreactive protein band in liver microsomes, with an apparent molecular size above 50 kD, in Atlantic cod (Fig. 3A). By using 2D gel electrophoresis followed by immunoblotting, two immunoreactive CYP3A protein spots were detected above 50 kD, with pI values around 4.8 and 5.1, respectively (Fig. 3B). The most basic isoprotein appears to be inducible by treatment with ketoconazole (Fig. 3B). Ethynylestradiol and nonylphenol treatment did not induce expression of the more basic isoform. Present data does not elucidate whether those two protein spots are different gene products, or if they result from post-translational modifications such as phosphorylation.

### *In vitro *inhibition studies

*In vitro *inhibition studies using pooled Atlantic cod liver microsomes showed that ketoconazole, nonylphenol, ethynylestradiol and the ketoconazole:nonylphenol (1:5) mixture inhibited CYP1A (EROD) activity, with IC_50 _values (inhibitor concentration required to achieve a 50% inhibition) ranging from 0.6 to 20 μM. The CYP3A-mediated BFCOD activity also was inhibited by ketoconazole (IC_50 _= 0.3 μM), ethynylestradiol (IC_50 _= 40 μM) and the ketoconazole:nonylphenol (1:5) mixture (IC_50 _= 5:25 μM). Nonylphenol alone was an insignificant inhibitor of microsomal CYP3A activities in Atlantic cod (IC_50 _= 160 μM). For comparison, IC_50 _values for nonylphenol and ethynylestradiol also were determined in cDNA expressed human CYP3A4 baculovirus supersomes, compared to the prototypical CYP3A4 inhibitor ketoconazole (IC_50 _= 0.4 μM). In contrast to Atlantic cod liver microsomes, nonylphenol inhibited the human CYP3A4 mediated BFCOD activity (IC_50 _= 35 μM) and ethynylestradiol was a weak inhibitor (IC_50 _= 50 μM) of this activity. The IC_50 _values are summarized in Table 1.

**Table 1:** IC_50 _values and inhibition constants (K_i_) for ketoconazole and xenoestrogens on CYP1A- and CYP3A activities assayed *in vitro*.

**Compound(s)**	**IC_50_(μM)^1,a^**	**K_i_(μM)^1,a^**	**IC_50_(μM)^2,b^**	**K_i_(μM)^2,b^**	**IC_50_(μM)^3,b^**
Ketoconazole (KC)	0.6 (0.0)^c^	0.04 – low [S]	0.3 (0.1)^c^	0.2	0.4^c^
		0.2 – high [S]			
Nonylphenol (NP)	5.2 (1.1)	3.5	160 (40)	Not analysed	35
Ethynylestradiol	20 (1.2)	5.4 – low [S]	40 (7.1)	54 – low [S]	50
		10.3 – high [S]		95 – high [S]	
KC:NP (1:5)	1.3 (0.2):6.2 (1.0)	Not analysed	5.3 (1.1):25.0 (5.3)	Not analysed	Not analysed

^1 ^Hepatic Microsomal CYP1A activity; ^2 ^Hepatic Microsomal CYP3A activity; ^3 ^cDNA Expressed Human CYP3A4; ^a ^Substrate [S] = 7-Ethoxyresorufin; ^b ^[S] = 7-Benzyloxy-4-[trifluoromethyl]-coumarin; ^c ^Published in [22]. Each IC_50 _value represents the mean from 2–4 separate assays, followed by the SD, in brackets. The K_i _values are estimated from one representative Dixon plot.

**Figure 4:**
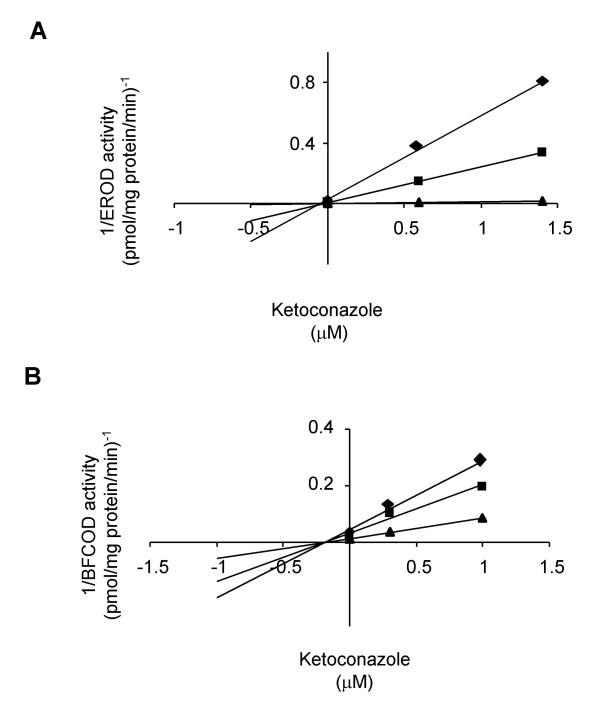
**Non-competitive inhibition of CYP1A by ketoconazole (A) and non-competitive inhibition of CYP3A by ketoconazole (B)**. Dixon plots for ketoconazole on A) EROD activity (diamonds represent 8.2; squares represent 25 and triangles represent 677 pM ethoxyresorufin). B) BFCOD activity (diamonds represent 48; squares represent 84 and triangles represent 200 μM BFC).

**Figure 5:**
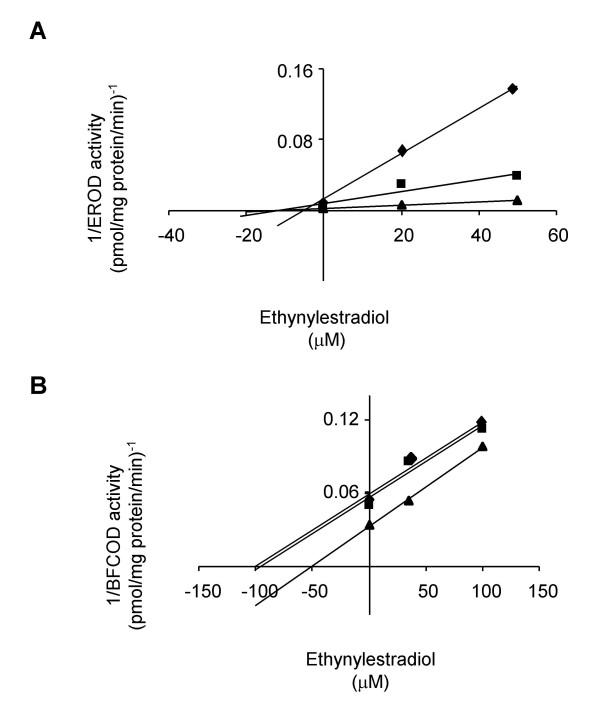
**Non-competitive inhibition of CYP1A by ethynylestradiol (A) and uncompetitive inhibition of CYP3A by ethynylestradiol (B)**. Dixon plots for ethynylestradiol on A) EROD activity (diamonds represent 8.2; squares represent 25 and triangles represents 677 pM ethoxyresorufin). B) BFCOD activity (diamonds represent 200; squares represent 267 and triangles represents 356 μM BFC).

**Figure 6:**
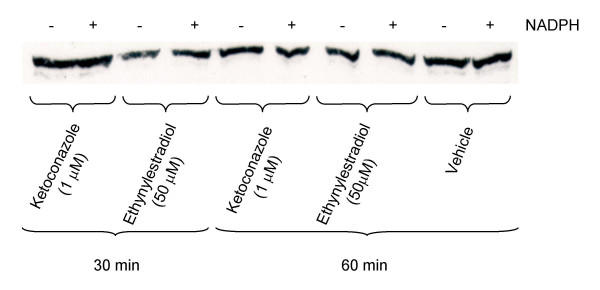
**CYP3A Western blot after *in vivo *incubation**. Western blot of CYP3A proteins in pooled liver microsomes from Atlantic cod detected using PAb against rainbow trout CYP3A. The blot illustrates representative samples after *in vitro *incubation with 1.0 μM ketoconazole and 50 μM ethynylestradiol for 30 or 60 min.

The inhibitory effects of these compounds were further investigated on hepatic microsomal CYP1A and CYP3A enzyme kinetics. The K_i _values were determined in Dixon plots (Figs. 4 and 5) and summarized in Table 1. Ketoconazole was a potent non-competitive inhibitor of both CYP1A and CYP3A activities with K_i _values in the sub-μM range (Fig. 4; Table 1). Ethynylestradiol was a non-competitive inhibitor of CYP1A with K_i _from 5.4 to 10.3 μM and an uncompetitive inhibitor of CYP3A with K_i _from 54 to 95 μM (Fig. 5; Table 1). Nonylphenol was a non-competitive inhibitor of CYP1A activity with K_i _around 3.5 μM (Table 1). There were no effects of pre-incubation either with ketoconazole or ethynylestradiol on hepatic microsomal CYP3A protein levels in this study (Fig. 6).

### Plasma vitellogenin- and sex steroid hormone levels

Treatment with nonylphenol, ethynylestradiol and the combination of ketoconazole and nonylphenol resulted in induction of vitellogenin, whereas these treatments had no statistically significant effect on 17β-estradiol, testosterone and 11-keto-testosterone plasma levels compared to either vehicle treated fish or fish treated with each test compound alone. The results are summarized in Table 2.

**Table 2:** Plasma levels of vitellogenin and sex steroid hormones in juvenile Atlantic cod exposed *in vivo *to ketoconazole and xenoestrogens.

**Treatment**	**Vitellogenin**	**17β-Estradiol**	**Testosterone**	**11-Keto-Testosterone**
	(μg/ml plasma)	(pg/ml plasma)	(pg/ml plasma)	(pg/ml plasma)

	n = 7–8	n = 8	n = 7–8	n = 6–8

Vehicle (5 ml peanut oil/kg b.w.)	0.6 (1.0)	62 (56)	86 (68)	33 (38)
Ketoconazole (KC) (12 mg/kg b.w.)	0.6 (0.9)	60 (26)	120 (78)	71 (46)
Nonylphenol (NP) (25 mg/kg b.w.)	266 (199) ^a^	76 (52)	75 (33)	42 (67)
Ethynylestradiol (5 mg/kg b.w.)	4,350 (1,463) ^a^	100 (69)	90 (40)	35 (23)
KC + NP (12 + 25 mg/kg b.w.)	268 (205) ^a^	36 (28)	61 (51)	36 (38)

^a ^Significantly different from vehicle treated fish (*P *< 0.001; Kruskal-Wallis ANOVA, followed by Mann-Whitney U-test). Each value represents the mean from 6–8 fish, followed by the SD, in brackets.

## Discussion

### Effects on CYP1A

For data evaluation we must bear in mind that western blot analysis of CYP1A protein levels is less sensitive than the EROD assay [41] and so densitometry analysis of western blot data fails to detect minor changes. Treatment of juvenile Atlantic cod with ketoconazole resulted in elevated EROD activities. Mixed exposure to ketoconazole and nonylphenol resulted in induced EROD activities and CYP1A protein levels. Induction of hepatic CYP1A gene expression by exposure to imidazoles and/or triazoles also has been reported in rat, bobwhite quail (*Colinus virginianus*) and rainbow trout (*Oncorhynchus mykiss*) [8,13,37,38]. However, it is possible that induction of EROD activity, partly or completely, is masked by CYP1A inhibition caused by ketoconazole present in the tissue. Inhibition of CYP1A is supported in the present study, showing that ketoconazole was a potent non-competitive inhibitor of EROD activity *in vitro*. Ketoconazole and other imidazoles also have been shown to be potent inhibitors of EROD activities in other vertebrates [9,13,14,42].

Treatment of Atlantic cod with nonylphenol and ethynylestradiol resulted in decreased EROD activities, whereas no effects of these substances were observed on CYP1A protein levels. This decrease in EROD activity is probably caused by nonylphenol or ethynylestradiol present in the liver microsome fraction. Nonetheless, chemical data are required, in the future, to confirm this. *In vitro *inhibition studies in liver microsomes confirmed that nonylphenol and ethynylestradiol acted as non-competitive inhibitors of the EROD activity. Hence, ketoconazole, nonylphenol, and ethynylestradiol interact with CYP1A enzymes, indicating a possible site for interaction of these different classes of xenobiotics. In addition, ketoconazole treatment induces CYP1A expression, which further may affect this interaction.

### Effects on CYP3A

Atlantic cod exposed to nonylphenol, ethynylestradiol and ketoconazole displayed reduced hepatic CYP3A (BFCOD) activities. The CYP3A inhibitory effect by ketoconazole is well known and ketoconazole is the most established diagnostic inhibitor, used to assess human *in vitro *CYP3A4 activity [12,43]. Studies in fish demonstrate that ketoconazole is a potent inhibitor of hepatic BFCOD activities in killifish (*Fundulus heteroclitus*), rainbow trout and Atlantic cod with IC_50 _values at 0.01, 0.1 and 0.3 μM, respectively [13,22]. In rainbow trout, exposure to ketoconazole resulted in elevated hepatic and intestinal CYP3A protein levels [13]. In the present study, 2D gel electrophoresis revealed the presence of two CYP3A immunoreactive spots in Atlantic cod liver microsomes with pI values around 4.8 and 5.1, respectively. The more basic isoform (pI 5.1) appeared to be responsive to ketoconazole treatment. The existence of multiple CYP3A genes has been shown in several vertebrate species, including teleosts [44]. It is conceivable that there are two different CYP3A genes in Atlantic cod and that these genes respond differently to ketoconazole treatment. Protein isoforms revealed on 2D gel electrophoresis may also be due to post-translational modifications such as phosphorylation [45]. Phosphorylation of several members of the CYP2 gene family, through phosphokinase A, resulted in immediate loss in catalytic activity [46]. The shift to a more basic form in this report could imply a dephosphorylation of CYP3A upon ketoconazole treatment. However, as these spots were not detected directly on the 2D gels by using either Coomassie blue or silver staining, no spots could be selected for sequencing to investigate whether these two immunoreactive spots represent different gene products.

In juvenile Atlantic salmon (*Salmo salar*), multiple hepatic CYP3A proteins also were seen [19]. The two proteins responded differently to nonylphenol treatment. High doses of nonylphenol (125 mg/kg b.w.) suppressed the high-molecular weight CYP3A protein band, whereas lower doses of nonylphenol (25 mg/kg b.w.) resulted in induction of this isoform [19]. In the present study, exposure to nonylphenol resulted in reduced CYP3A activities in juvenile Atlantic cod liver. Nevertheless, nonylphenol did not inhibit microsomal BFCOD activities *in vitro*, whereas nonylphenol was a weak inhibitor of that activity using recombinant human CYP3A4. The Atlantic cod we exposed to a mixture of ketoconazole and nonylphenol displayed *in vivo *CYP3A activities that were lower than the additive effect of each compound administered alone. The mechanism for this possible interaction still is not known. In mammals, more than one substrate can simultaneously bind to the active site of CYP3A4 [11]. Thus, in Atlantic cod, conceivably both ketoconazole and nonylphenol might bind to CYP3A enzyme and prevent access of the diagnostic BFC substrate. The CYP3A protein levels remained unchanged in these fish suggesting that combined exposure of ketoconazole and nonylphenol selectively inhibits *in vivo *CYP3A activity.

Ethynylestradiol has been shown to act as a mechanistic inactivator (*i.e. *"suicide" substrate) of the CYP3A4 enzyme, resulting in loss of CYP3A4 protein levels [47,48]. In Atlantic cod, a possible mechanism-based inactivation of CYP3A by ethynylestradiol was suspected. Thus, exposure to ethynylestradiol resulted in significantly reduced CYP3A levels and ethynylestradiol acted as an uncompetitive inhibitor of microsomal CYP3A activities. However, pre-incubation of hepatic microsomes with ethynylestradiol for up to 60 min did not result in any significant loss of CYP3A protein content, which implies that, in Atlantic cod, ethynylestradiol is not acting as mechanism-based inhibitor of CYP3A. Nonetheless, further studies are needed, as for example 2D gel electrophoresis of pre-incubated liver microsomes followed by immunoblotting.

### Vitellogenesis and sex steroid hormones

In humans, prolonged ketoconazole therapy results in decreased clearance of 17β-estradiol, which may cause gynecomastia, presumably through inhibition of hepatic CYP3A4 [5]. In the present study, nonylphenol dependent induction of vitellogenesis was not significantly affected by treatment with a single dose of ketoconazole. In the present study, exposure to xenoestrogens and ketoconazole alone had no statistically significant effect on sex steroid levels compared to control fish. In another study in first spawning Atlantic cod, exposure to alkylphenols resulted in decreased plasma levels of 17β-estradiol (in females) and testosterone and 11-keto-testosterone (in males) [49]. Additional data, including plasma levels of ethynylestradiol, and increasing the sample sizes are required to definitely elucidate whether, in Atlantic cod, exposure to xenoestrogen and ketoconazole alone or in combination may affect sex steroid homeostasis.

## Conclusions

This study identifies, in Atlantic cod, interactions between ketoconazole and two different types of xenoestrogens on CYP1A and CYP3A. Ketoconazole acted as a non-competitive inhibitor of CYP1A and CYP3A activities and ketoconazole treatment also induced CYP1A protein expression. Ethynylestradiol acted as a non-competitive inhibitor of CYP1A and an uncompetitive inhibitor of CYP3A activities. *In vitro *studies revealed that nonylphenol was a non-competitive inhibitor of CYP1A; but it did not inhibit CYP3A. However, *in vivo*, nonylphenol synergistically impaired the ketoconazole-mediated inhibition of CYP3A activity, without affecting CYP3A protein levels. The study further illustrates that induction of CYP1A- and CYP3A gene expression can be partly or completely masked by inhibition of catalytic activities or vice versa. Taken together, the results indicate that CYP1A and CYP3A represent sites of interactions between those classes of xenobiotics. In future risk-assessment of, *e.g.*, municipal effluents or produced water from oil platforms, that have been shown to contain xenoestrogens, it should be considered to identify other classes of substances, for example azoles that also interact with CYP1A and CYP3A. Our data may warn for ecotoxicological implications, as induction of EROD activity as well as plasma vitellogenin routinely are used as biomarkers to assess exposure to AHR and ER agonists in various biomonitoring programs in the aquatic environment.

## Methods

### Chemicals

The 4-nonylphenol and the 17α-ethynylestradiol, for the *in vivo *exposure experiment, were obtained from Fluka Chemie AG (Buchs, Switzerland). The 4-nonylphenol for the *in vitro *inhibition studies was from Berol Nobel (Stenungsund, Sweden). Dimethylsulphoxide (DMSO), 7-ethoxyresorufin, horseradish peroxidase- (HRP) conjugated goat-anti-mouse IgG, iodoacetamide, ketoconazole, ponceau-*S*, resorufin and tween-20 were obtained from Sigma Aldrich (Stockholm, Sweden). Reduced nicotinamide-adenine-dinucleotide-phosphate (NADPH) was from Roche Diagnostics (St Louis, MO, USA and Bromma, Sweden). Ready gels (12% continuous acrylamide in Tris:HCl), 3-[(3-cholamidopropyl)-dimethylammonio]-1-propanesulfonate (CHAPS), precision protein standards (low range) and supported nitrocellulose membrane (0.45 μm) were purchased from BioRad (Sundbyberg, Sweden). The 17β-estradiol and testosterone enzyme immuno assay (EIA) kits were purchased from Cayman Chemical (Ann Arbor, MI, USA). The 11-keto-testosterone EIA kit and the Atlantic cod vitellogenin Enzyme Linked ImmunoSorbent Assay (ELISA) kit were obtained from Biosense Laboratories AS (Bergen, Norway). HRP-conjugated donkey-anti-rabbit IgG, the ECL™ Western blotting detection reagents and Immobiline™ DryStrip 7 cm ranging from pH 4 to 7 were from Amersham Biosciences (Uppsala, Sweden). Ampholytes for isoelectric focusing (Servalyt^® ^Carrier ampholyt 3–10) was purchased from Serva Feinbiochemica (Heidelberg, Germany). Dithiothreitol (DTT), Kodak X-Omat AR-ray film, X-ray developer and fix were from VWR International (Stockholm, Sweden). The 7-benzyloxy-4-[trifluoromethyl] coumarin (BFC), 7-hydroxy-4-[trifluoromethyl] coumarin (HFC) and the CYP3A4 inhibition kit were from BD Biosciences Company, Gentest™ (Woburn, MA, USA). All other chemicals used were of the purest grade available in Sweden or Norway, from Sigma-Aldrich, BioRad and VWR international.

### Animals and sampling

Hatchery reared juvenile Atlantic cod of both sexes with an average body weight (b.w.) around 400 g were supplied by Sekkingstad, Preserving AS, Hordaland, Norway. The fish were kept in 0.5 m^3 ^indoor glass fibre tanks, at Industrial Laboratory (ILAB), Bergen High Technology Centre (Bergen, Norway), provided with continuously flowing seawater at a temperature of 8 ± 0.5°C and a salinity of 3.4%. Throughout the experimental period, the fish were subjected to continuous 24 h artificial light (the regime the farm used for optimal fish growth). The fish were acclimated to these conditions for five days prior to the experimental period. During the experimental period the fish were starved and i.p. injected with either 12 mg ketoconazole/kg b.w. resuspended in peanut oil (2.5 mg/ml); 25 mg nonylphenol/kg b.w. dissolved in peanut oil (5.0 mg/ml); 5 mg ethynylestradiol/kg b.w. dissolved in peanut oil (1.0 mg/ml) or a mixture of ketoconazole and nonylphenol (12 mg ketoconazole + 25 mg nonylphenol/kg b.w. in peanut oil). Control fish were injected with 5 ml peanut oil/kg b.w. (vehicle). There were eight to nine fish in each treatment group. When designing the experiment, we could only test one combination due to limited fish numbers. We selected nonylphenol to combine with ketoconazole because a previous study showed that, in Atlantic cod, alkylphenols affect CYP1A/3A more strongly than the natural estrogen 17β-estradiol [22]. The ketoconazole dose (12 mg/kg) was selected based on the results on CYP1A and CYP3A protein levels and enzyme activities from a previous dose-response study in rainbow trout [13]. The nonylphenol dose (25 mg/kg) was selected as this dose is known to induce vitellogenesis in a number of fish species. In addition, in a previous study on the Atlantic salmon, this dose of nonylphenol also had effects on CYP1A and CYP3A [19].

After five days exposure, the fish were sacrificed by a sharp blow to the head. Blood samples were collected from the dorsal vein using a heparinized syringe and the liver was quickly dissected out and placed in ice-cold homogenization buffer (0.1 M sodium phosphate buffer pH 7.4, containing 0.15 M KCl, 1 mM EDTA and 1 mM DTT). Liver microsomes were prepared according to the published protocol by Goksøyr [50], and stored at -80°C. Total microsomal protein content was measured according to a published method by Bradford [51], using bovine serum albumin as standard, and a SpectraFluor spectrophotometer from Tecan (Grödig/Salzburg, Austria). Blood plasma was isolated by centrifugation at 5,000 g for 10 min at room temperature and stored at -80°C. Ethical approval licence number of ILAB Bergen: 119. Experiment no. 0204.

For *in vitro *inhibition studies, feral Atlantic cod of both sexes were caught off the West coast of Sweden and placed in concrete basins provided with recirculating aerated seawater at 10 ± 2°C and a salinity of 3.0% and alternative light/dark photoperiods of 12 hours. Prior to sampling, the animals were starved and acclimated to these conditions for three weeks. Eight fish were injected i.p. with β-naphthoflavone (BNF), 50 mg/kg b.w. dissolved in peanut oil (5.0 mg/ml). The fish were placed in a 100 l glass aquarium provided with aerated seawater (above) and 30% of the water volume was replaced each day. To eliminate visual stress, the sides of the aquaria were covered with black plastic sheets. After 3 days exposure, the fish were sacrificed. Livers were quickly dissected out and placed in ice-cold homogenization buffer. Livers were pooled from twenty untreated Atlantic cod and from eight BNF treated Atlantic cod, respectively. Microsomal fractions were isolated (above) and stored in aliquots at -80°C. Ethical approval from the Ethical committee of Göteborg license number (99–2003). The duration of exposure was decided according to results from previous time-course studies showing maximal CYP1A protein and EROD activities in rainbow trout and in the marine viviparous blenny (*Zoarces viviparous*), 3 days post-injection with either the prototypical CYP1A inducers BNF or 3-methylcholanthrene [52-54].

### CYP1A- and CYP3A protein blot analyses

Western blot analyses of 40 μg hepatic microsomal protein were carried out using enhanced chemoluminescence (ECL), based on the protocol previously described [55] and PAb raised against rainbow trout CYP1A and CYP3A [41,55,56]. The intensity of each protein band was determined by densitometry on scanned fluorograms using Labview 7.0 from National Instruments (Austin, TX, USA).

The 2D gel electrophoresis was performed using immobilised pH gradient gels with linear gradient from pH 4 to 7. The samples were concentrated by acetone precipitation and pellets dissolved in rehydration buffer (8 M urea, 2 M thiourea, 20 mM DTT, 4% CHAPS, 0.5% Triton X-100, 0.5% ampholyte 3–10 and <0.02% bromophenolblue) to a final protein concentration of 20 μg/μl or 80 μg/μl. The samples were rehydrated overnight followed by isoelectric focusing for 2.5 h. The rehydration was passive and carried out overnight in an Immobiline Dry Strip reswelling tray (Amersham Biosciences). First-dimension isoelectric focusing (IEF) was performed on a Multiphor II unit (Amersham Biosciences) at 20°C using a MultiDrive XL power supply (Pharmacia LKB). Settings for IEF were 30 min at 100 V and 3 h at 3500 V for a total of 10,520 Vh. Amperage and wattage were set to 2 mA and 5 W. The proteins were resolved on 9% continuous acrylamide gel in Tris:HCl, including sodium dodecyl sulphate polyacrylamide using a mini-gel apparatus from BioRad at 200 V for 45 min. Each sample consisted of pooled liver microsomes from eight to nine fish from each treatment group. Gels loaded with 25 μg microsomal protein were initially stained with 0.1 % (w/v) Coomassie brilliant blue, and then destained, followed by silver staining. The latter was performed according to Heukeshoven and Dernick [57]. Stained gels were scanned and analyzed using the PDQUEST 7.1 software (BioRad). Gels loaded with 100 μg microsomal proteins were electrotransferred to nitrocellulose membrane and immunoblotted for CYP3A, as described above.

### Catalytic assays

The CYP1A activity was determined as EROD activity, using resorufin as standard in a SpectraFluor plate reader according to the protocol provided by Nilsen *et al. *[58]. The CYP3A catalytic activity was measured as BFCOD activity, using HFC as standard. The BFC assay was performed based on a published protocol by Miller *et al. *[59] and optimized for rainbow trout liver microsomes (T. Hegelund and M. Celander, unpublished data). The reaction mixture consisted of 200 μM BFC, bovine serum albumin (1.6 mg/ml), 2 μM NADPH and 10 μl liver microsomes in a total volume of 200 μl in 0.2 M potassium phosphate buffer pH 7.4 in a 96-multiwell plate using a VICTOR™ 1420 Multilabel Counter from Wallac Sverige AB (Upplands Väsby, Sweden).

### *In vitro *inhibition of CYP1A and CYP3A

*In vitro *inhibition studies were carried out in 96-multiwell plates using a VICTOR™ 1420 Multilabel Counter. The IC_50 _values were determined for nonylphenol, ethynylestradiol, ketoconazole and the ketoconazole:nonylphenol (1:5) mixture on CYP1A and CYP3A activities. The substances were dissolved in DMSO and diluted with ethanol. The final concentrations never exceeded 0.01% (v/v) DMSO and 0.001% ethanol (v/v). For CYP1A and CYP3A inhibition studies, pooled liver microsomes from BNF-treated and from untreated Atlantic cod, respectively, were used. For comparison, the IC_50 _values for ketoconazole, nonylphenol and ethynylestradiol were determined in cDNA expressed human CYP3A4 baculovirus supersomes using the CYP3A4 inhibition kit from BD Gentest.

### *In vitro *incubation studies

Pooled liver microsomes from untreated Atlantic cod were pre-incubated for 10, 30 and 60 min, at room temperature, with ethynylestradiol and ketoconazole following CYP3A western blot analysis. The reaction mixture consisted of microsomes (2.5 or 5.0 mg protein/ml) and various concentrations of ethynylestradiol (35, 50 and 100 μM) or ketoconazole (0.3 and 1.0 μM) ± 3 μM NADPH in a total volume of 50 μl in homogenization buffer, containing 20% (v/v) glycerol. The CYP3A western blot analysis was performed as described above. Ethynylestradiol and ketoconazole were dissolved in acetonitrile (vehicle) and the final acetonitrile concentration in the reaction mixture was 0.02% (v/v).

### Plasma vitellogenin analysis

Plasma levels of vitellogenin protein were determined using a non-competitive sandwich ELISA kit and employing rabbit PAb against Atlantic cod vitellogenin from Biosense Laboratories AS (Bergen, Norway) [58]. Each plasma sample was diluted (1:20, 1:15,000 and 1:50,000) and 100 μl was analyzed and compared to purified Atlantic cod vitellogenin protein standards (ranging between 0.12 and 2,000 ng/ml). The signal was detected at A_492 _after 15 min incubation with substrate solution, using a VICTOR™ 1420 Multilabel Counter.

### Plasma sex steroid hormone analyses

Plasma levels of 17β-estradiol and testosterone were determined using competitive EIA kits from Cayman Chemical (Ann Arbor, MI, USA). Plasma levels of 11-keto-testosterone were analyzed using a competitive EIA kit, from Biosense Laboratories AS (Bergen, Norway). Plasma from each fish was concentrated (2:1) by extraction once with six volumes diethyl ether and 50 μl was analyzed and compared to purified standard substances. The signals were detected at A_405 _after 40 min (17β-estradiol), 60 min (testosterone) or 80 min (11-keto-testosterone) incubation with substrate solution, using a VICTOR™ 1420 Multilabel Counter.

### Statistics

Data were tested for homogeneity of variances using the Levene's test. When there was homogeneity of variances we used a parametric one-way ANOVA, followed by Scheffé post hoc test. When there was no homogeneity of variances we used the non-parametric Kruskal-Wallis ANOVA, followed by the two-tailed Mann-Whitney U test. No values were log transformed. Data are presented as means (n = 6–9 fish) accompanied with the standard deviations (SD). The significance level (α) was set at 0.05. The statistical analyses were performed using SPSS 11.0 software, from SPSS Sweden AB (Sundbyberg, Sweden).

## Authors' contributions

LH performed most of the analyses, participated in fish exposure, sampling, experimental design and drafted the manuscript. BEG assisted with experimental design, 2D-analysis and writing. AG participated in experimental design and writing. MCC rose funding, coordinated, participated in fish exposure, sampling, experimental design and writing.
